# Therapeutic Potential and Molecular Mechanisms of *Emblica officinalis* Gaertn in Countering Nephrotoxicity in Rats Induced by the Chemotherapeutic Agent Cisplatin

**DOI:** 10.3389/fphar.2016.00350

**Published:** 2016-10-03

**Authors:** Salma Malik, Kapil Suchal, Jagriti Bhatia, Sana I. Khan, Swati Vasisth, Ameesha Tomar, Sameer Goyal, Rajeev Kumar, Dharamvir S. Arya, Shreesh K. Ojha

**Affiliations:** ^1^Cardiovascular Research Laboratory, Department of Pharmacology, All India Institute of Medical SciencesNew Delhi, India; ^2^Department of Pharmacology, R. C. Patel Institute of Pharmaceutical Education and ResearchShirpur, India; ^3^Department of Pharmacology and Therapeutics, College of Medicine and Health Sciences, United Arab Emirates UniversityAl Ain, UAE

**Keywords:** inflammation, apoptosis, oxidative stress, *Emblica officinalis*, cisplatin, nephrotoxicity

## Abstract

*Emblica officinalis* Gaertn. belonging to family Euphorbiaceae is commonly known as Indian gooseberry or “Amla” in India. It is used as a ‘rejuvenating herb’ in traditional system of Indian medicine. It has been shown to possess antioxidant, anti-inflammatory and anti-apoptotic effects. Thus, on the basis of its biological effects, the present study was undertaken to evaluate the protective effect of the dried fruit extract of the *E. Officinalis* (EO) in cisplatin-induced nephrotoxicity in rats and also to evaluate the mechanism of its nephroprotection. The study was done on male albino Wistar rats. They were divided into six groups (*n* = 6) viz. control, cisplatin-control, cisplatin and EO (150, 300, and 600 mg/kg; p.o. respectively in different groups) and EO only (600 mg/kg; p.o. only). EO was administered orally to the rats for a period of 10 days and on the 7th day, a single injection of cisplatin (8 mg/kg; i.p.) was administered to the cisplatin-control and EO treatment groups. The rats were sacrificed on the 10th day. Cisplatin-control rats had deranged renal function parameters and the kidney histology confirmed the presence of acute tubular necrosis. Furthermore, there were increased oxidative stress, apoptosis and inflammation along with higher expression of MAPK pathway proteins in the rat kidney from the cisplatin-control group. Contrary to this, EO (600 mg/kg) significantly normalized renal function, bolstered antioxidant status and ameliorated histological alterations. The inflammation and apoptosis were markedly lower in comparison to cisplatin-control rats. Furthermore, EO (600 mg/kg) inhibited MAPK phosphorylation which was instrumental in preserving renal function and morphology. In conclusion, the results of our study demonstrated that EO attenuated cisplatin-induced nephrotoxicity in rats through suppression of MAPK induced inflammation and apoptosis.

## Introduction

Cisplatin (*cis*-diamminedichloroplatinum II or CDDP) is a platinum compound which has a broad role in the management of various solid malignancies such as head and neck, bladder, lung, ovaries, testicles, and uterus (Oh et al., 2016). However, during the course of chemotherapy, cisplatin may cause ototoxicity, nephrotoxicity, myelosuppression, and peripheral neuropathy (Oh et al., 2014). Among these undesirable effects, nephrotoxicity is the most important dose limiting adverse effect of cisplatin therapy, which primarily affects the S3 segment of the proximal tubules (Bolisetty et al., 2016). It accounts for up to 60% of acute kidney injury cases acquired from the hospital and is associated with remarkably high morbidity and mortality and approximately one fourth of patients initiated on high-dose cisplatin have severe renal dysfunction while one third experience kidney injury within few days of receiving cisplatin (Oh et al., 2016). Clinically, cisplatin induced renal dysfunction manifests as decline in renal plasma flow and glomerular filtration rate with an increase in serum creatinine and blood urea nitrogen (Miller et al., 2010). The proposed molecular mechanisms underlying cisplatin nephrotoxicity involve oxidative stress, DNA damage, apoptosis and exaggerated inflammatory response (Pabla and Dong, 2008).

The oxidative stress has emerged as the main mechanism in cisplatin-induced nephrotoxicity (dos Santos et al., 2012; Oh et al., 2016). It has been reported that excess reactive oxygen species (ROS) production as well as antioxidant system depletions are consequent to cisplatin administration (Domitrović et al., 2013). The likely sources of ROS during cisplatin administration include the mitochondrial electron transport chain system (Pan et al., 2015), xanthine oxidase (Yousef and Hussien, 2015), cytochrome P450 enzymes (Pabla and Dong, 2008), and NADPH oxidase (Wang et al., 2015). Since, ROS are highly reactive and unstable; they may attack and modify cellular components such as lipids, proteins, and DNA, resulting in cellular stress (Jaiman et al., 2013). ROS accumulation also activates important signaling pathways, including apoptotic pathway, which leads to cell death in the event of cisplatin-induced nephrotoxicity (Song et al., 2015). Cisplatin mediated oxidative stress provokes the stimulation of a cascade of signaling proteins, including MAPKs and NF-κB (Malik et al., 2015). This abnormal activation of MAPK and NF-κB leads to release of inflammatory cytokines subsequently aggravating the renal damage (Sahu et al., 2014). The renal damage induced by cisplatin is managed using hydration/diuretics, renal function monitoring and adjustment of the cisplatin dose. However, renal toxicity still occurs despite the use of these measures. Therefore, taking the molecular pathways of cisplatin nephrotoxicity in consideration, exploration of more effective nephroprotective therapeutic options which would not affect the tumoricidal activity of cisplatin is essential.

Several strategies have been evaluated to minimize the nephrotoxicity caused by cisplatin. Recently, plant derived natural compounds and their active constituents are being evaluated for their beneficial effects in various pathophysiological conditions (Athira et al., 2016). *Emblica officinalis* (*E. Officinalis*) also known as Amla or Indian Gooseberry is a natural fruit that serves as a rich source of vitamin C (Krishnaveni and Mirunalini, 2010). It also contains flavonoids, tannins, terpenoids and alkaloids which possess various biological activities (Kim et al., 2005). The active extracts of *E. Officinalis* have been demonstrated to exert a wide range of activities such as antioxidant in alcohol-induced oxidative damage in rat liver microsomes (Reddy et al., 2014), anti-apoptotic in arsenic-induced apoptosis in thymocytes of mice (Singh et al., 2013), anti-inflammatory in rodent models of acute and chronic inflammation (Golechha et al., 2014), anti-diabetic in type 2 diabetes in rats (Nain et al., 2012), anti-hypertensive in DOCA salt induced hypertension in rats (Bhatia et al., 2011) and anticancer in cervical cancer cells (Mahata et al., 2013). Several studies have also been conducted to investigate its beneficial effect in kidney injury including cyclophosphamide induced renal injury (Haque et al., 2001) and mycotoxin induced renal toxicity (Verma and Chakraborty, 2008). Thus, the present study was designed to investigate the beneficial effect of the dried fruit extract of *E. officinalis* (EO) on cisplatin mediated kidney injury by assessing its antioxidant, anti-apoptotic, and anti-inflammatory properties on the kidney and further to evaluate whether MAPK pathway is involved in mediating this renoprotection.

## Materials and Methods

### Plant Extract

The dried fruit extract of *E. officinalis* (EO) was obtained from Sanat Products Limited, India (a WHO-GMP and ISO 9001 Accredited Herbal Extract Manufacturer Company). The batch number of EO was 048001. The extract contained 31.58% w/w of hydrolysable tannins emblicanin A and emblicanin B on dried weight basis.

### Chemicals

Cisplatin was obtained from Pfizer Products Pvt. Ltd., India. Blood urea nitrogen (BUN) and serum creatinine kits were procured from Transasia Bio-Medicals Ltd., India. Kits for Terminal deoxynucleotide transferase dUTP nick end labeling (TUNEL) assay (ApoBrduDNA fragmentation assay kit) (Biovision Inc. California), tumor necrosis factor-α (TNF-α) (Diaclone Tepnel Company, UK) and IL-6 (Ray Biotech, Inc. Norcross, GA, USA) were used. Primary antibodies against extracellular signal-regulated kinase 1/2 (ERK1/2), phospho-ERK1/2 (p-ERK1/2), c-Jun N-terminal kinase (JNK), phospho-JNK (p-JNK), Caspase-3, NF-κBp65, and β-actin were procured from cell signaling technology, USA. Antibodies for Bcl-2, Bax and p-38 were purchased from Abcam, UK. Phospho-p38 (p-p38) antibody was obtained from Santa Cruz biotechnology, USA. Secondary antibodies were from Merck Genei Pvt. Ltd., India. All other chemicals used were of analytical grade.

### Experimental Animals

Adult male albino Wistar rats aged 10–12 weeks old (weighing 150–200 g) were used for the study. All experimental procedures were conducted after approval by Institutional Animal Ethics Committee of All India Institute of Medical Sciences, New Delhi (IAEC no. 604/IAEC/2011) and conformed to the Indian National Science Academy Guidelines for care and use of animals in research. During the study period, animals were kept in polypropylene cages under conditions of ambient temperature (25 ± 2°C), relative humidity (60 ± 5%) and 12 h light/dark cycle and provided food pellets (Ashirwad Industries Ltd, Chandigarh, India) and tap water *ad libitum*.

A total of 36 male albino Wistar rats were randomly divided into six groups containing six rats per group. The test drug, EO, was dissolved in distilled water for administration to rats.

#### Group 1 (Control)

Rats received distilled water (2 ml/kg; p.o.) for the period of 10 days.

#### Group 2 (Cisplatin-Control)

Rats received distilled water (2 ml/kg; p.o.) for the period of 10 days and on the 7th day, a single injection of cisplatin (8 mg/kg; i.p.) was administered to induce nephrotoxicity (Malik et al., 2015).

#### Groups 3–5 (Treatment Groups)

Rats received EO (150–600 mg/kg; p.o.) daily for the period of 10 days and on the 7th day, a single injection of cisplatin (8 mg/kg; i.p.) was administered to induce nephrotoxicity.

#### Group 6 (EO Only)

Rats received EO (600 mg/kg; p.o.) for the period of 10 days.

On the 10th day, rats were anaesthetized with pentobarbitone sodium (60 mg/kg; i.p.) and chest cavity was opened. Blood was drawn *via* cardiac puncture, and centrifuged at 4000 rpm to separate serum which was stored at -20°C for measurement of BUN, serum creatinine, TNF-α and IL-6 levels. After sacrificing, one kidney from 6 rats per group was snap frozen in liquid nitrogen and preserved in -80°C and used for biochemical estimation. The second kidney from 3 rats per group was removed and preserved in 10% neutral buffered formalin for histopatholgical and TUNEL assay and kidney from the other 3 rats per group were snap frozen in liquid nitrogen and stored in -80°C for western blot analysis.

### Assessment of Kidney Function Parameters

Serum creatinine and BUN levels were measured using respective commercially available kits.

### Measurement of Renal Oxidant–Antioxidant Parameters

Kidney sample was removed from -80°C, thawed, weighed and 10% homogenate was prepared in ice-chilled phosphate buffer (0.1 M, pH 7.4). The tissue homogenate was divided into two parts. One part of this homogenate was used for the estimation of malondialdehyde (MDA) level and reduced glutathione (GSH) content. The other part of this tissue homogenate was centrifuged at 5000 rpm and supernatant thus obtained was used for the estimation of superoxide dismutase (SOD), catalase (CAT) enzyme activities and protein content.

#### Estimation of MDA Level

MDA level was estimated by the method described by Ohkawa et al. (1979). To the 0.1 ml tissue homogenate, 0.5 ml of 8.1% sodium dodecyl sulfate, 1.5 ml of 20% acetic acid and 1.5 ml of 0.8% thiobarbituric acid was added, vortexed and heated at 95°C for 60 min. After cooling, 5 ml of butanol:pyridine (15:1) mixture was added to extract pink color complex. The pink organic layer was separated and absorbance read at 532 nm. The amount of MDA was calculated by extrapolation from the MDA standard curve and expressed as nmole/g tissue.

#### Estimation of GSH Content

GSH was measured by the method described by Moron et al. (1979). Firstly, homogenate was centrifuged with an equal volume of 10% trichloroacetic acid. To the 0.1 ml of supernatant, 3 ml Na_2_HPO_4_ (0.3 M; pH 8.0) and 0.5 ml of 5,5′-dithiobis (2-nitrobenzoic acid) prepared in 1% trisodium citrate were added and mixed thoroughly. The absorbance of yellow color formed was read at 412 nm. The amount of GSH was determined by extrapolation from standard curve and expressed as μmole/g tissue.

#### Estimation of SOD

Superoxide dismutase enzyme activity was determined by the method described by Marklund and Marklund (1974). To the 0.1 ml supernatant, 2.95 ml of phosphate buffer (0.1 M; pH 8.4) and 0.05 ml of pyrogallol (7.5 mM) was added and the change in absorbance was recorded at an interval of 60 s for 2 min at 420 nm. One unit of enzyme activity was defined as the amount of enzyme required to produce 50% inhibition of pyrogallol autoxidation under assay conditions and expressed as U/mg protein.

#### Estimation of CAT

CAT enzyme activity was estimated by the method described by Aebi (1984). To the 0.05 ml of supernatant, 1 ml of phosphate buffer (50 mM; pH 7.0) and 1.0 ml of hydrogen peroxide (H_2_O_2_) was added. Immediately, thereafter, change in the absorbance was recorded for 30 s at an interval of 5 s at 240 nm. One unit of CAT enzyme activity is equal to 1 μmol of H_2_O_2_ decomposed per min and expressed as U/mg protein.

#### Estimation of Protein Content

Protein was measured by a method described by Bradford (1976). To the supernatant, Bradford reagent was added and vortexed. Blue color formed was measured at 595 nm. The amount of protein was determined by standard curve and expressed as mg/ml.

### Measurement of Serum Pro-inflammatory Cytokines Level

TNF-α and IL-6 levels were assessed in serum using commercially available ELISA kits as per manufacturer instructions.

### Histopathological Examination

For histopathological evaluation, paraffin blocks were made from kidney tissue preserved in formalin. The tissue sections of 5-μm thickness were cut using microtome (Leica RM 2125, Germany). These sections were stained with hematoxylin and eosin (H&E) and studied under light microscope (Dewinter Technologies, Italy).

### TUNEL Assay

TUNEL assay was performed for detection of apoptosis in the renal tissue. TUNEL assay was performed according to the method described by Malik et al. (2015). Briefly, sections were incubated with Proteinase K for 30 min to enhance tissue permeability and then treated with 30% H_2_O_2_ in methanol for 15 min to diminish any endogenous peroxidase activity. Later, sections were incubated with complete labeling reaction buffer and antibody solution, each for 1 h and 30 min. Following this, 3,3′-diaminobenzidine (DAB) solution was added and at least five fields in each slide were checked for any TUNEL positive cells in each group.

The pathologist evaluating histopathological and TUNEL slides was blinded to the treatment groups.

### Western Blot Analysis

For western blot analysis, kidneys were removed from -80°C, thawed and weighed. Then tissues were homogenized in Radioimmunoprecipitation assay (RIPA) buffer (150 mM NaCl, 10% Triton X-100, 0.5% Sodium deoxycholate, 0.1% Sodium dodecyl sulfate, 50 mM Tris base), along with protease inhibitor (Sigma Aldrich, USA). The homogenate was centrifuged at 12000 rpm for 20 min at 4°C and supernatant was used for measurement of protein concentration by using the method described by Bradford (1976). Protein equivalent to 40 μg was separated by the sodium dodecyl sulfate polyacrylamide gel electrophoresis (SDS-PAGE). Separated proteins were then transferred to a nitrocellulose membrane and then blocked with 3% bovine serum albumin (BSA) for 1 h. After that, membrane was blocked with primary antibodies for ERK1/2, p-ERK1/2, JNK, p-JNK, p38, p-p38, Bcl-2, Bax, Caspase-3, NF-κBp65 and β-actin (1:3000) overnight at 4°C. The primary antibodies were detected with HRP-conjugated secondary antibodies (1:5000) for 2 h at room temperature. The antigen-antibody reaction was then visualized with enhanced chemiluminscence (ECL) kit according to manufacturer’s instructions. The band intensity was measured using image-j software.

### Statistical Analysis

Data of all experimental groups were analyzed by one way analysis of variance (ANOVA) followed by *post hoc* Tukey-Kramer multiple comparison test using the Graph Pad InStat software. Data are expressed as mean ± SEM and values for *P* < 0.05 were considered as statistically significant.

## Results

### Effect of EO on Kidney Function Parameters

The kidney function parameters such as serum creatinine and BUN levels were measured in all treatment groups to assess renal function. Cisplatin injection resulted in significant increase in serum creatinine (*P* < 0.001) and BUN levels (*P* < 0.001) in comparison to control group. This elevation in serum creatinine and BUN levels suggests significant kidney damage and confirmed the induction of nephrotoxicity in cisplatin control group. EO (600 mg/kg) pretreatment significantly (*P* < 0.01) normalized serum creatinine and BUN levels as compared to cisplatin-control group. However, no significant effect was observed at two lower doses (150 and 300 mg/kg) (**Table 1**).

**Table 1:** Effect of EO on renal function tests (serum creatinine and BUN) and biochemical parameters.

Groups	Sr. Creatinine (mg/dl)	BUN (mg/dl)	MDA (nmol/g tissue)	GSH (μmol/g tissue)	SOD (U/mg protein)	CAT (U/mg protein)
Control	0.57 ± 0.028	28.59 ± 1.62	60.50 ± 3.44	0.29 ± 0.012	3.98 ± 0.41	4.73 ± 0.42
Cis-C	1.45 ± 0.052^∗∗∗^	53.95 ± 2.67^∗∗∗^	109.81 ± 3.75^∗∗∗^	0.16 ± 0.014^∗∗∗^	2.42 ± 0.33^∗∗^	2.36 ± 0.65^∗∗^
EO 150+Cis	1.37 ± 0.075^†††^	50.35 ± 1.72^†††^	102.94 ± 4.54^†††^	0.18 ± 0.015^†††^	2.91 ± 0.18	3.61 ± 0.53
EO 300+Cis	1.29 ± 0.048^†††^	46.01 ± 2.05^†††^	96.63 ± 4.63^†††^	0.21 ± 0.012^††^	3.02 ± 0.19	4.13 ± 0.30
EO 600+Cis	1.18 ± 0.057^†††##^	42.46 ± 1.66^†††##^	91.66 ± 3.38^†#^	0.24 ± 0.014^##^	3.57 ± 0.15^#^	4.42 ± 0.26^#^
EO 600 only	0.62 ± 0.031	31.65 ± 1.35	71.01 ± 3.71	0.32 ± 0.012	3.96 ± 0.18	4.92 ± 0.33

*BUN, blood urea nitrogen; MDA, malondialdehyde; GSH, reduced glutathione; SOD, superoxide dismutase; CAT, catalase. Data are expressed as mean ± SEM of 6 rats per group. ^∗∗^*P* < 0.01, ^∗∗∗^*P* < 0.001 versus control group; ^†^*P* < 0.05, ^††^*P* < 0.01, ^†††^*P* < 0.001 versus control group; ^#^*P* < 0.05, ^##^*P* < 0.01 versus Cis-C group.*

### Effect of EO on Renal Oxidant–Antioxidant Parameters

In cisplatin-control rats, there was a significant (*P* < 0.001) increase in the level of MDA, a marker of lipid peroxidation, along with reduction in the level of antioxidants GSH (*P* < 0.001), SOD (*P* < 0.01), and CAT (*P* < 0.01) in comparison to control group. Interestingly, EO (600 mg/kg) pretreatment significantly restored the kidney antioxidant status. This is depicted by increase in the activities of GSH (*P* < 0.01), SOD (*P* < 0.05) and CAT (*P* < 0.05) and decrease in the level of MDA (*P* < 0.05) as compared to the cisplatin-control rats. Moreover, the two lower doses (150 and 300 mg/kg) of EO failed to exert any beneficial effect on the kidney antioxidant status (**Table 1**).

### Effect of EO on Serum Inflammatory Cytokines Level

Cisplatin administration is known to release pro-inflammatory cytokines in serum. Thus, there was a significant (*P* < 0.001) increase in serum pro-inflammatory cytokines level (TNF-α and IL-6) in the cisplatin-control group as compared to the rats in the control group. EO (600 mg/kg) pretreatment significantly (*P* < 0.01) prevented the increase in the serum cytokines level as compared to the cisplatin-control group. However, no significant difference in the levels of these cytokines was observed with the lower doses of EO (150 and 300 mg/kg) (**Table 2**).

**Table 2:** Effect of EO on serum pro-inflammatory cytokines level.

Groups	TNF-α (pg/ml)	IL-6 (pg/ml)
Control	37.35 ± 1.49	9.5 ± 0.46
Cis-C	58.17 ± 2.38^∗∗∗^	17.92 ± 0.91^∗∗∗^
EO 150+Cis	54.95 ± 2.92^†††^	16 ± 0.90^†††^
EO 300+Cis	50.07 ± 1.87^††^	14.21 ± 0.94^††^
EO 600+Cis	46.46 ± 1.84^†##^	12.62 ± 0.65^†##^
EO 600 only	38.51 ± 1.64	10.29 ± 0.65

*TNF-α, tumor necrosis factor-α; IL-6, interleukin-6. Data are expressed as mean ± SEM of 6 rats per group. ^∗∗∗^*P* < 0.001 versus control group; ^†^*P* < 0.05, ^††^*P* < 0.01, ^†††^*P* < 0.001 versus control group; **^##^***P* < 0.01 versus Cis-C group.*

### Effect of EO on Renal Histopathology

**FIGURE 1:**
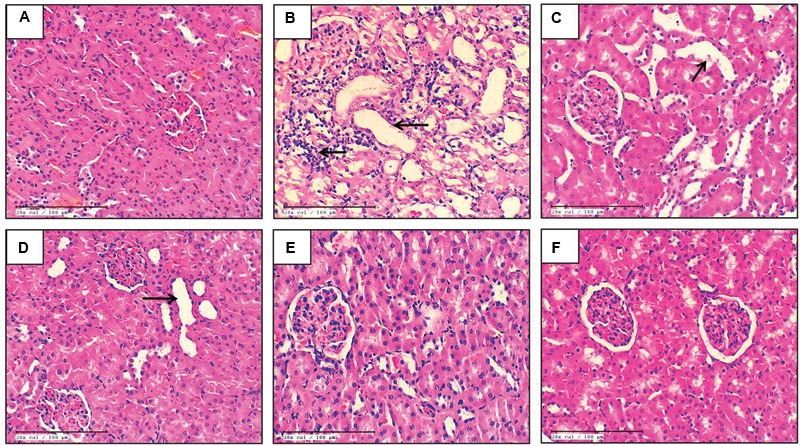
**Light microscopic study (H&E) of renal tissue in various experimental groups. (A)** Control; **(B)** Cis-C; **(C–E)** EO 150, 300, 600 mg/kg+Cis respectively; **(F)** EO 600 mg/kg only. (→): acute tubular necrosis; (*n* = 3; 20X; scale bar 100 μm).

The histopathological evaluation of control and EO only groups demonstrated normal architecture of tubules with no evidence of inflammation (**Figures 1A,F**). The kidney sections from the cisplatin-control rats showed tubular atrophy, denudation of epithelium and infiltration of inflammatory cells (**Figure 1B**). In the groups, pretreated with 150 and 300 mg/kg dose of EO, there was marked and moderate tubular damage and inflammation respectively (**Figures 1C,D**). However, the highest dose of EO i.e., 600 mg/kg exerted significant nephroprotection and a marked absence of tubular necrosis and inflammation in the kidneys was observed (**Figure 1E**).

Thus, on the basis of results of the above mentioned parameters, EO at the dose of 600 mg/kg was found to exert maximum nephroprotection. Hence, this dose was used for further TUNEL and western blot analysis.

### Effect of EO on Apoptosis and Inflammation

**FIGURE 2:**
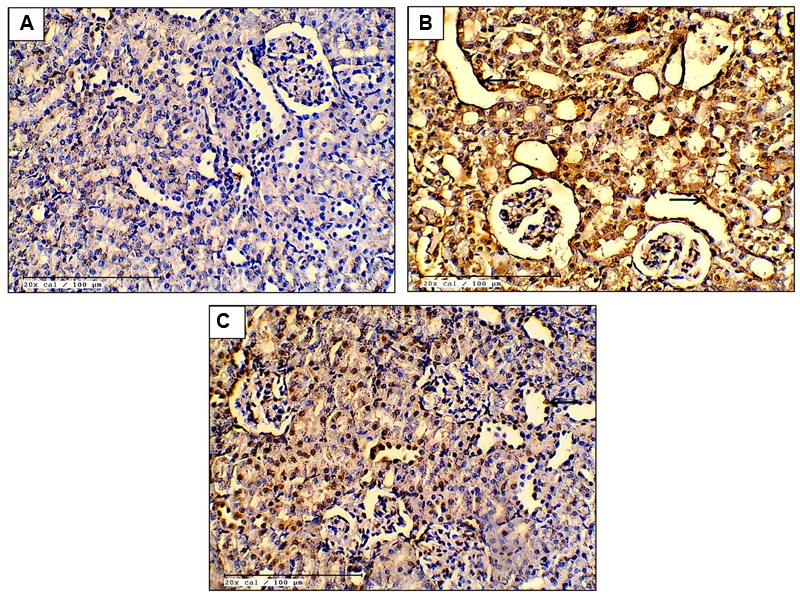
**Effect of EO on TUNEL positivity (**A–C**; 20X; scale bar 100 μm) in various experimental groups. (A)** Control; **(B)** Cis-C; **(C)** EO 600 mg/kg+Cis. (→): tubular cell apoptosis.

**FIGURE 3:**
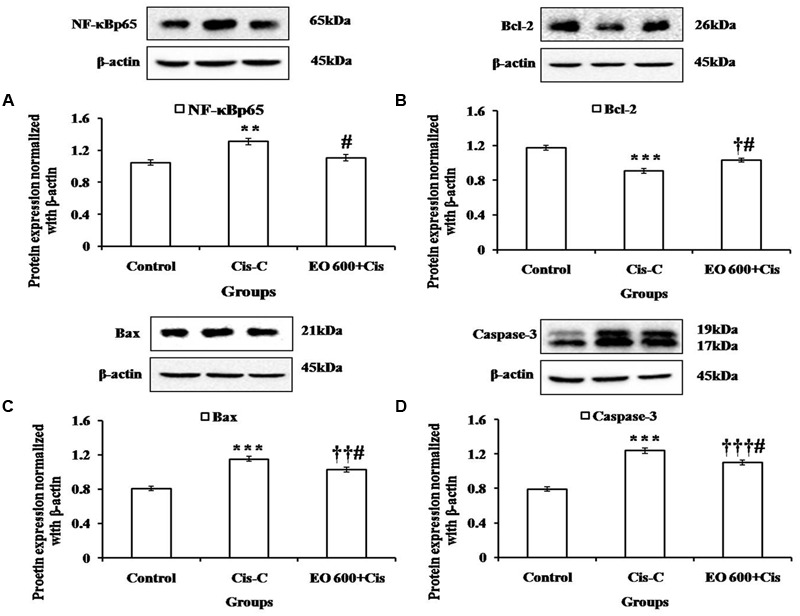
**Effect of EO on **(A)**: NF-κBp65; **(B)** Bcl-2; **(C)** Bax; **(D)** Caspase-3 levels in various experimental groups.** Data are expressed as mean ± SEM of 3 rats per group. ^∗∗^*P* < 0.01, ^∗∗∗^*P* < 0.001 versus control group; ^†^*P* < 0.05, ^††^*P* < 0.01, ^†††^*P* < 0.001 versus control group; ^#^*P* < 0.05 versus Cis-C group.

The apoptosis in the renal tissue was assessed by detecting the expression of apoptotic proteins in all groups. In cisplatin-control group, there was significantly increased expression of pro-apoptotic proteins [Bax (*P* < 0.001) and Caspase-3 (*P* < 0.001)] and decreased expression of Bcl-2 (*P* < 0.001), an anti-apoptotic protein. Furthermore, there was increased DNA fragmentation and increased number of TUNEL positive cells in the cisplatin-control group as compared to the control group. However, EO (600 mg/kg) treatment group reduced the apoptosis as there was significantly (*P* < 0.05) increased expression of Bcl-2 and decreased expression of Bax, Caspase-3 along with decreased DNA fragmentation as compared to cisplatin-control group. This confirms the anti-apoptotic effect of EO in renal tissue (**Figures 2** and **3**).

Further, western blot analysis was performed to determine the expression of NF-κBp65 in the renal tissue. Cisplatin-control rats demonstrated significantly (*P* < 0.01) increased level of NF-κBp65 whereas treatment with EO (600 mg/kg) attenuated this effect (*P* < 0.05) (**Figure 3**).

### Effect of EO on MAPK Signaling Pathway

**FIGURE 4:**
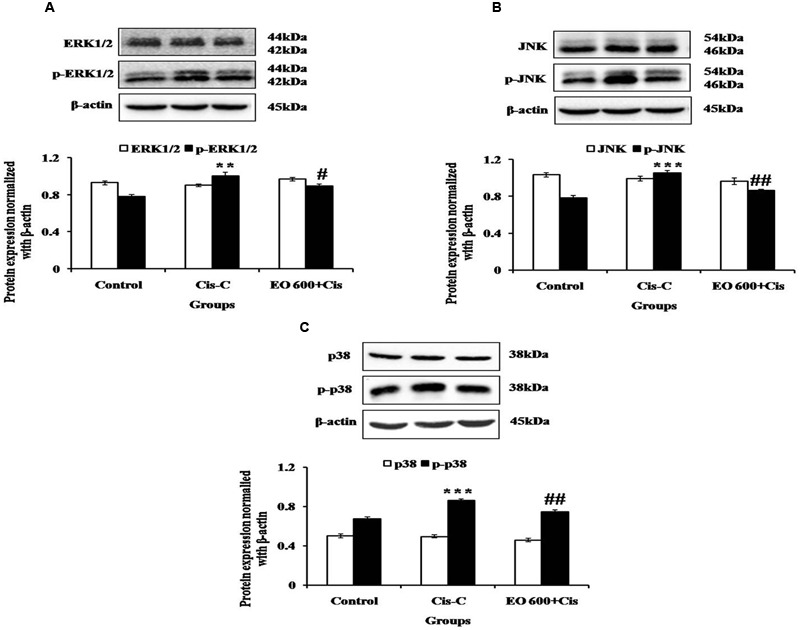
**Effect of EO on **(A)** ERK1/2 and p-ERK1/2; **(B)** JNK and p-JNK; **(C)** p38 and p-p38 in various experimental groups.** Data are expressed as mean ± SEM of 3 rats per group. ^∗∗^*P* < 0.01, ^∗∗∗^*P* < 0.001 versus control group; ^#^*P* < 0.05, ^##^*P* < 0.01 versus Cis-C group.

In cisplatin-control group, there was increased phosphorylation of ERK1/2, JNK and p38 proteins as compared to control rats. The increased phosphorylation of these proteins mediated apoptosis and inflammation in the cisplatin-control group. Contrary to this, EO (600 mg/kg) treatment halted the activation of this pathway and prevented apoptosis and inflammation in the renal tissue (**Figure 4**).

## Discussion

The current study has demonstrated the renoprotective effect of EO in cisplatin-induced acute renal toxicity in rats. The administration of EO virtually ameliorated most of the deleterious effects of cisplatin. This renoprotection was evident from improved functional as well as structural renal profiles, blunting of oxidative stress, inflammation, and apoptosis. Our results further provided evidence that this improvement was mediated by suppression of the MAPK signaling cascade.

Cisplatin’s chemotherapeutic applicability is limited by renal toxicity and the latter occurs due to accumulation of cisplatin into the renal tubular cells which subsequently leads to renal tubular cell injury and renal cell death which appears histologically as tubular atrophy (Pabla et al., 2009). Many studies have proposed that cisplatin is also injurious to renal vasculature which results in decreased blood flow leading to ischemic injury of the kidneys, appearing as a decline in the glomerular filtration rate which is reflected as increased serum creatinine and BUN levels (Xu et al., 2015). In our study, we observed increased levels of serum creatinine and BUN suggesting renal damage due to cisplatin. Serum creatinine is the waste metabolic product and is formed due to breakdown of creatine phosphate in muscle. Normally, it is excreted by the kidneys, primarily by glomerular filtration but also by proximal tubular secretion with little or no reabsorption. If there is deterioration in kidney function, serum level of creatinine rises (El-Naga, 2014; Changizi-Ashtiyani et al., 2016; El-Naga and Mahran, 2016). Also, concomitant decline in urinary creatinine clearance is observed vis a vis raised serum creatinine levels. Infact, decrease in urinary creatinine clearance due to cisplatin-induced kidney injury has been reported by other researchers (Quesada et al., 2012; Nojiri et al., 2015). Although, we have not measured the urinary creatinine clearance in this particular study, the changes in serum creatinine and BUN levels observed here were clearly accompanied by changes in renal histology, further suggesting renal function deterioration in cisplatin-treated animals. There was marked renal tubular atrophy and denudation of epithelium following intraperitoneal administration of 8 mg/kg cisplatin. Administration of EO 600 mg/kg, significantly normalized serum creatinine and BUN levels and also preserved the histology of renal tubular cells.

Numerous studies have depicted the key role of oxidative stress in the pathophysiology of cisplatin-induced renal cell death (Song et al., 2015). Once cisplatin reaches the tubular cells, it rapidly reacts with thiol-containing molecules including glutathione by being converted to a highly reactive electrophile. This positively charged electrophile is generated by replacement of the chloride ligands of cisplatin with water molecules. Cisplatin also increases ROS synthesis by inducing mitochondrial dysfunction and disrupting the electron transport chain. Due to their unstable configuration, ROS reacts with membrane lipids, cellular proteins and DNA resulting in their modification leading to cellular stress (Pabla and Dong, 2008). Lipid peroxidation is a consequence of excessive ROS production. It leads to increased level of MDA (a lipid peroxidation marker). The rise in MDA levels following cisplatin administration *in vivo* has been observed in our study and has also been reported by other workers (Yüce et al., 2007). Excessive ROS is tackled by endogenous antioxidants. However, when the synthesis of ROS overrides its destruction, there is overconsumption of these antioxidants. This consumption has also been demonstrated previously in cisplatin nephrotoxicity models (Al-Majed et al., 2006). Similarly, there was consumption of glutathione and other endogenous antioxidants such as SOD and CAT in the cisplatin-control group of our study. However, pretreatment with EO maintained glutathione and other antioxidants at near normal levels in the renal tissue. This supports the antioxidant activity of EO which has been documented in the past (Golechha et al., 2012; Dutta and Sahu, 2013; Singh et al., 2014). Previous studies have shown that active constituents such as emblicanins A and B, gallic acid, and ellagic acids present in *E. officinalis* are responsible for its antioxidant activity (Feeney, 2004). Furthermore, free radical scavenging property of *E. officinalis* has been reported to be near to that of L-ascorbic acid, a well known antioxidant (Muthuraman et al., 2011).

Though oxidative stress is a known promoter of apoptosis, cisplatin itself directly induces apoptotic cell death which has been shown by *in vitro* and *in vivo* studies (Tsuruya et al., 2003; Seth et al., 2005; Zhou et al., 2013). According to past reports, renal tubular epithelial cell apoptosis induced by cisplatin is primarily *via* the mitochondrial pathway (Liu et al., 2014). Cisplatin induced apoptosis has been shown to be regulated by the pro-apoptotic protein Bax and the anti-apoptotic protein Bcl-2 (Jiang et al., 2009). It has been shown that cisplatin induces activation of Bax genes (Wei et al., 2007). Bax protein eventually undergoes a conformational change and binds to mitochondrial membrane and subsequently causes the release of cytochrome c from mitochondria leading to apoptosis (Tayem et al., 2006). In contrast, the anti-apoptotic protein Bcl-2 stabilizes the mitochondrial membrane potential thereby inhibiting cytochrome c release and inhibiting apoptosis (Cummings and Schnellmann, 2002). Therefore, Bax and Bcl-2 are crucial in regulating apoptosis. In our experiment, significantly higher Bax and Caspase-3 and lower Bcl-2 levels were observed in cisplatin-control group. This shows that cisplatin increases Bax and Caspase-3 while lowering Bcl-2 expression of renal tissues whereas pretreatment with EO (600 mg/kg) decreased the Bax and Caspase-3 and increased Bcl-2 levels. TUNEL assay was performed to assess the DNA fragmentation. The TUNEL positivity was observed to be high in cisplatin treated rats and significantly reduced in EO pretreated group. These findings support the previously documented anti-apoptotic activity of *E. Officinalis* (Singh et al., 2013, 2014). Thus, it can be proposed that the renoprotection of EO is mediated by its antioxidant and anti-apoptotic properties.

Strong evidence suggests that the pathogenesis of cisplatin induced renal cell apoptosis is associated with the release of inflammatory cytokines and mediators, including IL-1, IL-6, and TNF-α (Zhang et al., 2007; Guerrero-Beltrán et al., 2012; Zirak et al., 2014). Additionally, NF-κB which is a transcription factor regulating the modulation of inflammatory and immunomodulatory genes, is associated with the process of cisplatin induced renal inflammation (Benedetti et al., 2013). NF-κB activity is inhibited by the specific IκB in the cytoplasm, the latter being rapidly cleared by IKKβ upon activation of NF-κB. NF-κB is released and then translocates to the nucleus, where it activates the transcription of target genes (Ma et al., 2015). Our study has shown that cisplatin-control group has a higher expression of activated NF-κB, IL-6 and TNF-α in comparison to the control rats. Interestingly, EO significantly attenuated the levels of inflammatory cytokines and also blunted the expression of NF-κB. The polyphenol rich components of ethanolic extract of *E. officinalis* have previously been demonstrated to reduce expression of NF-κB in a similar fashion (Kim et al., 2010). Therefore, the attenuation of cisplatin induced nephrotoxicity with EO in our study may also be mediated by the anti-inflammatory property of EO.

Based on the ability of EO to suppress the phosphorylation of members of the NF-κB cascade pathway and inflammation, we further investigated the effect of EO on the upstream signaling components of NF-κB, the MAPK family. MAPK family comprises of three major serine/threonine kinase proteins such as ERK 1/2, JNK and p38 which are associated with cell growth and differentiation, and are extensively linked to inflammation, apoptosis and cell death. Several *in vitro* and *in vivo* studies have demonstrated the role of p38 and JNK in cisplatin-induced nephrotoxicity (Ramesh and Reeves, 2005, 2006; Francescato et al., 2007). ERK1/2 promotes apoptosis by decreasing the production of cytochrome c, phosphorylation of caspase-3, and accelerating the translocation of Bax from cytosol to mitochondria during cisplatin-induced renal cell death (Kim et al., 2014). Cisplatin-induced phosphorylation of JNK induces inflammation of the kidney tubules, apoptosis, and kidney dysfunction (Ma et al., 2015). As anticipated, in our study, cisplatin injection increased the phosphorylation of p38, ERK1/2 and JNK. Pre-treatment with EO, however, ameliorated this increase. Our results are also in line with our study which has demonstrated ERK1/2 modulated effect of *E. officinalis* (Sumitra et al., 2009). Hence, we can convincingly propose that EO mediates its nephroprotective action by regulating MAPK signaling pathway.

In summary, our findings demonstrate that EO treatment alleviates the cisplatin-induced cytotoxicity in kidney through suppressing the ROS mediated activation of MAPKs and NF-κB signaling cascades. Furthermore, EO treatment inhibits the synthesis of intracellular inflammatory cytokines and mediators. Our study suggests that EO has good potential and may be further evaluated clinically for treating cisplatin-induced nephrotoxicity.

## Author Contributions

SM, KS, and RK: Perform and analyze all the experiments. SK, SV, AT, and SG: Writing of the manuscript. JB, DA, and SO: Designed the study.

## Conflict of Interest Statement

The authors declare that the research was conducted in the absence of any commercial or financial relationships that could be construed as a potential conflict of interest.

The reviewers AI and JU and handling Editor declared their shared affiliation, and the handling Editor states that the process nevertheless met the standards of a fair and objective review.
